# The role of artificial intelligence in advancing scoliosis care: a rapid review of current evidence and future opportunities

**DOI:** 10.3389/fmed.2026.1774697

**Published:** 2026-02-24

**Authors:** Merce Avellanet, Judith Sanchez-Raya, Maria Chiara Maccarone, Yannis Dionyssiotis

**Affiliations:** 1 Rehabilitation Department, Pediatric Development Unit, Hospital Nostra Sra. de Meritxell, University of Andorra, Escaldes-Engordany, Andorra; 2 Physical Medicine and Rehabilitation Service, Hospital Campus Vall Hebron, Autonomus University of Barcelona, Barcelona, Spain; 3 Department of Neuroscience, Physical and Rehabilitation Unit, University of Padova, Padova, Italy; 4 2^nd^ Physical Medicine and Rehabilitation Department, National Rehabilitation Center EKA, Athens, Greece

**Keywords:** adolescent, artificial intelligence, idiopathic scoliosis, machine learning, systematic review

## Abstract

**Background:**

Adolescent idiopathic scoliosis (AIS), is a complex three-dimensional deformity of the spine that affects a significant percentage of the adolescent population. Its progressive nature and evolution variability complicates therapeutic decisions, generating the need for more accurate tools for diagnosis, prediction of risk of progression and optimization of treatments. Artificial intelligence (AI) and machine learning (ML) emerge as tools with significant potential for comprehensive management of AIS. Despite the enthusiasm for these applications, there are important limitations that need to be addressed. The aim of this rapid review is to address a timely synthesis of available research and assess the quality of published reviews.

**Methods:**

Systematic reviews and meta-analyses published by April 2025 in English on scoliosis and any intervention involving AI were included. Search was performed in Embase, Cochrane Review Database and Pubmed/medline using the terms MesH scoliosis idiopathic and intelligence artificial and systematic reviews and meta-analysis. Two independent reviewers screened titles and abstracts following the PRISMA RR guidelines and assessed full text articles with the AMSTAR 2 tool. Any disagreement was resolved by a third reviewer.

**Results:**

Five systematic reviews met inclusion criteria. 55% of the included studies used AI algorithms with convolutional neural networks, artificial neural networks, decision trees, support vector machines, and hybrid models. The main applications in AIS were automatic Cobb angle measurement with high accuracy (< 3° in some models), curve type classification, prediction of curve progression and patient education and clinical decision support using language models.

**Conclusion:**

AI offers promising solutions for AIS management, particularly in automated Cobb angle measurement and progression prediction. Combining deep learning models with clinical data may transform future practice, but external validation and clinical integration must be strengthened to enable effective implementation.

## 1 Introduction

In recent years, artificial intelligence (AI) has emerged as a transformative tool in the field of healthcare, with increasing applications in automating clinical tasks such as radiological analysis, disease progression prediction, and classification of clinical conditions (1). Within the domain of musculoskeletal disorders, AI has shown promise in the management of adolescent idiopathic scoliosis (AIS), a complex spinal deformity that typically manifests during adolescence and requires accurate assessment and longitudinal monitoring (2).

The expanding body of literature on AI applications in AIS reflects growing interest in enhancing clinical decision-making and streamlining workflows. With the growing availability of scientific literature to both healthcare professionals and the general public, critically evaluating reviews has become increasingly important. The rapid AI development presents challenges regarding both the benefits and potential risks associated with these technologies. To address this, a timely synthesis of recent findings is essential.

This rapid review aims to critically synthesize recent evidence on clinical applications of AI in AIS care. Specifically, it focuses on evaluating reported benefits, potential harms, and future perspectives discussed in published reviews, since reviews are used extensively for clinical and policy decisions. Unlike traditional systematic reviews, rapid reviews abbreviate or limit certain steps of the review process—such as the breadth of the literature search or the number of reviewers involved—in order to expedite evidence synthesis (3). In this case, our review concentrates on key clinical outcomes and the implications of AI use in AIS diagnosis, monitoring, and management from reviews since systematic reviews are subject to a range of biases (3, 4). Systematic reviews can be the basis of important practice and policy decisions; therefore, their appraisal is highly recommended. Without careful evaluation, there is a risk of accepting biased, incomplete or low-quality evidence, potentially leading to misinformation or poor healthcare decisions.

## 2 Materials and methods

According to rapid review methodology, we selected 3 information sources likely to retrieve relevant literature. We used PRISMA for Rapid Review (PRISMA-RR) checklist to define search and protocol (5).

### 2.1 Search strategy and criteria

We performed a search using PubMed via National Library of Medicine, Embase via Embase, and Cochrane reviews via Cochrane Library; from January 2010 to April 2025. The search strategy was based on Medical Subject Heading (MeSH) for PubMed and Entree for Embase, terms or equivalent using idiopathic scoliosis AND intelligent artificial OR Machine learning AND Systematic reviews OR Metanalysis.

The inclusion criteria were female and male with AIS diagnosis or suspicion (population); Use of AI tools or Machine Learning models (intervention); current standard of care (comparator or control); and outcomes focused on following benefits of AI for (i) diagnosis, (ii) treatment, (iii) follow-up, (iv) quality of life/psychological effects, and (v) possible harms associated with the use of AI (outcome). Studies were systematic reviews, meta-analysis, narrative reviews, scoping reviews, published in English between 01 January 2010 and 01 April 2025.

The exclusion criteria applied were articles not written in English, studies that did not include exclusively paediatric populations, those addressing other types of scoliosis or spinal deformities, and publications limited to conference abstracts, letters to the editor, or responses.

### 2.2 Data extraction and quality assessment

Data extraction was conducted independently by two reviewers using a tested and standardized form. For quality assurance and search strategy peer review, we validated the primary search strategy by testing if known relevant records were retrieved. We used the AMSTAR 2 (A MeaSurement Tool to Assess systematic Reviews) to assess the quality of systematic reviews, including those based on non-randomized studies of healthcare (4). Conflict between reviewers was either resolved between reviewers or by a third reviewer.

## 3 Results

### 3.1 Selection process

**Figure 1:**
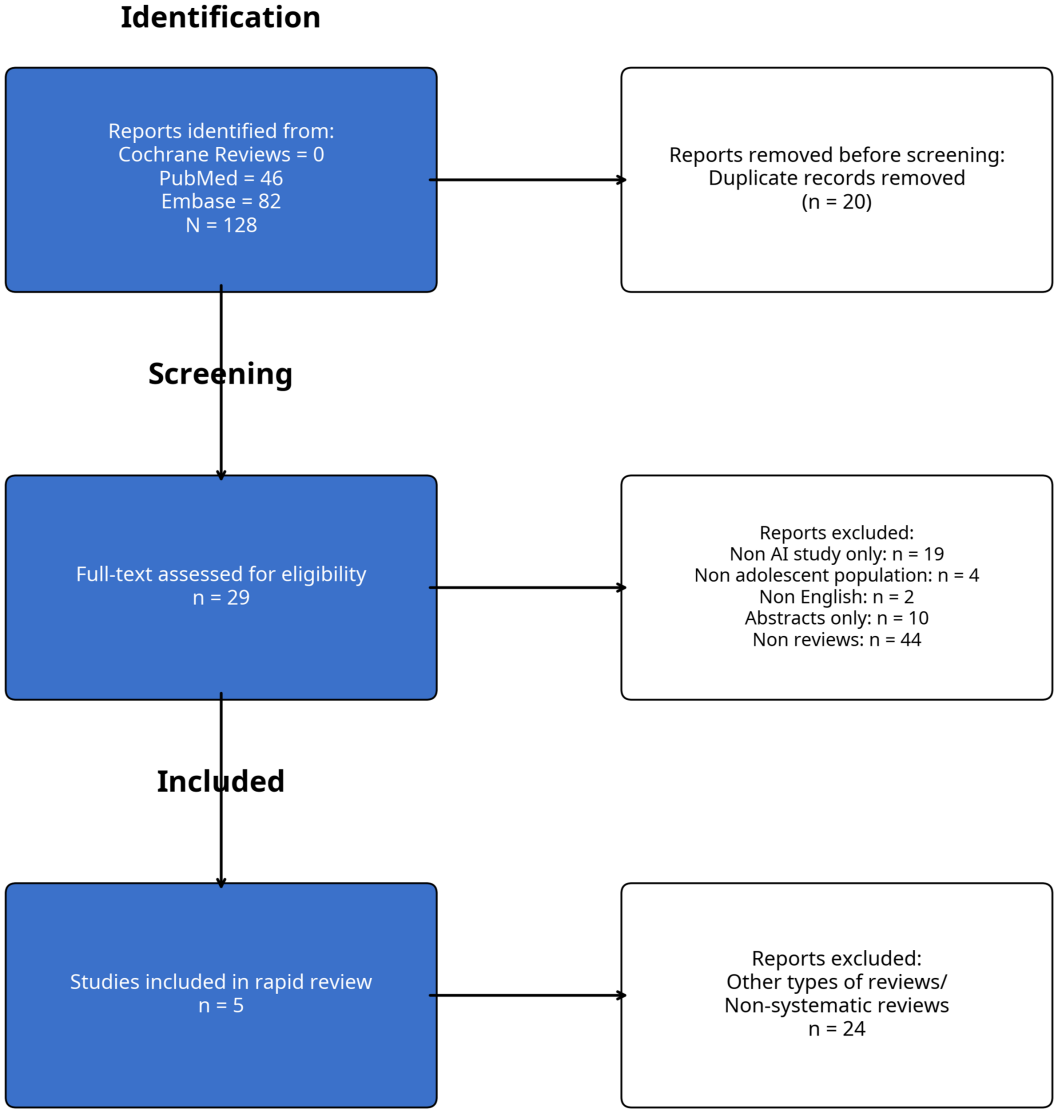
Literature review: PRISMA diagram.

The initial literature search retrieved 128 articles from Embase, Cochrane and PubMed. Following the abstract screening, 20 duplicate articles were identified and removed. Continuing with the predefined search strategy, a total of 29 full-text studies were reviewed (Figure 1). After analyzing these 29 articles, only five studies were independently selected by both reviewers to minimize potential selection bias (6–10). The two reviewers independently agreed that these five articles met the initial inclusion criteria, as they were systematic reviews with meta-analyses focusing on artificial intelligence and idiopathic scoliosis.

### 3.2 Quality assessment

Critical appraisal of reviews was independently performed by two reviewers using the 16 items of the AMSTAR 2 tool (Table 1) (4). This tool allows to evaluate reviews that include randomized or non-randomized studies of health care intervention. Conflict between reviewers was either resolved between reviewers or by a third reviewer.

**Table 1:** AMSTAR 2: critical appraisal tool for systematic reviews.

Item	Chen et al. (6)	Goldman et al. (7)	Zhang et al. (8)	Li and Wong (9)	Zhu et al. (10)
1- Research questions /inclusion criteria /components of PICO	N	PY	N	Y	Y
2- Protocol: explicit statement review methods established prior to the conduct of the review	N	Y	N	Y	Y
3- Explanation of the study designs included	PY	Y	Y	Y	Y
4- Comprehensive literature search strategy	N	Y	N	Y	Y
5- Study selection in duplicate	NS	Y	N	Y	Y
6- Data extraction in duplicate	NS	Y	N	NS	Y
7- List and justification of exclusion	N	Y	N	N	Y
8- Details of included studies	Y	Y	Y	Y	Y
9- Techniques for assessing risk of bias	N	N	N	N	Y
10- Sources of funding reporting	Y	Y	Y	N	Y
11- If meta-analysis, appropriate methods for statistical combination of results	NA	NA	NA	NA	Y
12- If meta-analysis, potential impact of RoB assessed	NA	NA	NA	NA	Y
13- Accounting for RoB when interpreting/ discussing results	N	N	NA	NA	Y
14- Satisfactory explanation for heterogeneity observed in results	Y	NA	N	Y	Y
15- If quantitative synthesis, adequate investigation of publication bias and its impact on results	NA	NA	NA	N	Y
16- Report potential sources of conflict of interest	Y	Y	N	N	Y

Y, YES; N, NO; PY, Partial YES, NA, Not applicable, NS, Not Speci!ed. PICO, Population Intervention Comparison Outcome & Timeframe.

Following the AMSTAR recommendations, an overall rating of confidence in the results of the published reviews are displayed in Table 2 (4). Two reviews were rated as low confidence in their results (6, 8), and two as moderate confidence in their results (7, 9). The metanalysis by Zhu et al. (10) was assessed as providing high-confidence results.

**Table 2:** Rating overall confidence in the results of review.

AMSTAR	Chen et al. (6)	Goldman et al. (7)	Zhang et al. (8)	Li and Wong (9)	Zhu et al. (10)
High					X
Moderate		X		X	
Low	X		X		
Critical low					

### 3.3 Characteristics of the included reviews

Chen K et al. review (6) was one of the first to describe how Machine Learning (ML), an advanced branch of AI, was driving a revolution in the clinical practice of scoliosis. It can impact every stage, from screening and diagnosis to surgical planning and prognosis prediction. For instance, ML algorithms, including Artificial Neural Networks (ANNs) and Convolutional Neural Networks (CNNs), significantly enhance early detection using images, reducing reliance on radiation and automating Cobb angle measurements. In surgical decision-making and assistance, ML helps classify curve types, identify optimal fusion regions, and supports precise pedicle screw insertion and 3D vertebral reconstruction. Furthermore, ML can predict postoperative complications like proximal junctional failure and surgical site infections and forecasting long-term patient outcomes. Despite its vast potential, the authors discuss several challenges including data limitations, issues with generalization, and the “black box” nature of some algorithms. Crucially, ethical concerns regarding patient data privacy and security must be addressed. Finally, the authors conclude that the future aims for a **“**human-and-machine” approach with multidisciplinary collaboration to fully integrate ML reliably into scoliosis care.

In 2023, Zhang H et al. (8) reviewed the impact of AI—utilizing ML and deep learning (DL)—on the diagnosis and treatment of scoliosis. AI significantly improves early imaging-based screening by enhancing diagnostic accuracy and reducing radiation exposure, including applications involving Large Language Models (LLMs) for patient education and severity assessment. It enables automatic and precise evaluation of spinal parameters, such as the Cobb angle, thereby reducing manual workload. In therapeutic decision-making, AI facilitates rapid classification, progression prediction, and surgical planning, including outcome forecasting. In surgical assistance, AI supports accurate pedicle screw placement and robot-assisted procedures, improving precision and reducing complications. Additionally, AI algorithms can effectively predict postoperative prognosis and potential complications. Despite these benefits, limitations remain, including data scarcity, limited generalizability, and critical ethical concerns related to patient privacy and algorithmic bias. The authors concluded similarly—that future efforts should focus on improving generalizability and ensuring the robust and ethical integration of AI into clinical practice.

Two additional reviews were published in 2024. One, authored by Goldman SN et al. (7) offers a comprehensive examination of the applications of A in the management of AIS. This review specifically explores the utilization of AI—particularly CNNs—in the automated analysis of radiographic images, classification of curve severity, and prediction of curve progression in AIS. The authors underscore the considerable potential of AI to enhance diagnostic accuracy and inform treatment decisions. However, they also identify several critical challenges, including the absence of standardized clinical implementation guidelines, limited model interpretability, and a lack of robust external validation. The review concludes by emphasizing the necessity of future research aimed at addressing these limitations to foster clinician trust and facilitate the effective integration of AI into routine clinical practice.

In the second one from 2024, Li L et al. (9) present a systematic review that highlights the ways in which various ML models—such as Random Forests (RF), Diffusion-Convolutional Neural Networks (DCNNs), Capsule Neural Networks (CapsNets), and Recurrent Neural Networks (RNNs) with Long Short-Term Memory (LSTM) units—enhance predictive accuracy compared to traditional approaches. These models leverage multidimensional input data, including patient demographics, clinical parameters, and two-dimensional imaging modalities (e.g., radiographs, surface topography scans, and smartphone-acquired photographs). The application of ML facilitates early imaging-based screening, automates measurement processes, and supports therapeutic decision-making by predicting the risk of curve progression, thereby enabling timely interventions or minimizing unnecessary radiation exposure. While these technologies offer promising avenues for personalized management of adolescent idiopathic scoliosis, several limitations persist. These include issues related to model interpretability (the so-called “black box” problem), the need for larger and more diverse multi-center validation studies, and the necessity of addressing key ethical concerns, particularly those involving patient data privacy.

The systematic review recently published by Zhu Y et al. (10) investigate DL algorithms for automated Cobb angle measurement on X-rays. The study aimed to provide an overview of DL algorithms, identify their limitations, and summarize possible solutions for improving their performance in scoliosis assessment. The Cobb angle is the gold standard for scoliosis diagnosis; however, manual measurement is time-consuming and prone to observer errors, highlighting the need for efficient automated tools. The researchers conducted a comprehensive literature search across six databases, ultimately including 50 studies in the systematic review and 17 in the meta-analysis. The meta-analysis, which primarily used the circular mean absolute error (CMAE) as the evaluation metric, reported an overall CMAE estimate of 2.99 (95% CI: 2.61–3.38). A significant finding was that segmentation-based methods demonstrated greater accuracy (CMAE 2.40) compared to landmark-based methods (CMAE 3.31). Despite high heterogeneity among the included studies, DL algorithms showed relatively high accuracy in automated Cobb angle measurement. The study also summarized potential strategies for improving model design in future research, such as using two-stage models, combining segmentation and landmark methods, or incorporating multi-view X-ray information. However, despite their accuracy, automated Cobb angle estimation methods are not yet widely adopted in routine clinical practice. The authors conclude that DL algorithms are promising, with segmentation-based methods potentially outperforming landmark-based approaches but emphasize the need for standardization to enable broader clinical adoption.

Table 3 summarized the years screened, articles included and scope of each of the five reviews.

**Table 3:** Summary of systematic reviews included.

Review	Years screened	Articles included	Scope
Chen et al. (6)	Screening, diagnosis and classification 2000–2021	21	Screening, diagnosis and classification
	Intraoperative manipulation 2005–2020	9	Intraoperative manipulation
	Complications prediction of surgery 2016–2020	9	Complications prediction of surgery
	Prognosis prediction and rehabilitation 2015–2019	6	Prognosis prediction and rehabilitation
Goldman et al. (7)	1985–2023	40	Development use or validation of AI models for diagnosis, treatment and prognosis
Zhang et al. (8)	Not specified (references range: 2000–2023)	58	Image screening Evaluation of scoliosis-related parameters Therapeutic decision-making Surgical assistance Prediction of prognosis
Li and Wong (9)	2016–2024	15	Curve prediction for nonsurgical patients with AIS
Zhu et al. (10).	Until sept 2023	50 of which 17 for meta-analysis	Deep leaning models for measuring Cobb angle on XRays, validated by clinical expert

## 4 Discussion

AIS is a complex three-dimensional spinal deformity with a reported prevalence between 0.47 and 5.2% (11). The diagnosis and treatment of AIS largely depend on accurate Cobb angle measurement and the prediction of curve progression (12).

In recent years, AI has emerged as a promising tool to automate clinical tasks such as radiological analysis, progression prediction, and curve classification (2, 13). Given the rapid expansion of literature on AI applications in AIS, this rapid review aims to critically synthesize current clinical applications and future perspectives reported in recent studies.

Systematic reviews and meta-analyses constitute the highest tier of scientific evidence. As scientific publications become increasingly accessible to both healthcare professionals and the public, the critical appraisal of review articles has become more important than ever. At the same time, the rapid development of AI poses challenges in understanding both its potential benefits and associated risks. Although AI holds considerable promise for enhancing diagnostic precision and guiding therapeutic decision-making in AIS, the findings of this rapid review do not substantiate a definitive clinical benefit or establish the reliability of AI applications in AIS management.

Chen K, et al. describe how ML can be a transformative technology in clinical treatment practice (6). They detail how it is applied at various stages of screening, diagnosis and classification, surgical decision making, intraoperative manipulations, complication prediction, prognosis prediction and manipulation.

In the scoping review of Goldmand et al. (7), the authors focus on AI applications in general, with 77.5% of the studies included published in the last 5 years, thus indicating the rapid growth of this field. The most common applications focus on automatic measurement of Cobb angle, axial vertebral rotation, and curve classification/severity. A major finding is the lack of clear clinical implementation guidelines (62.5% of studies), model transparency, and external validation. The “black box” nature of AI hinders clinician trust and generalizability. Other barriers include regulatory, financial, and ethical concerns.

Zhang et al. published of the more extensive reviews retrieved for our rapid review (8). They present multiple AI applications in scoliosis, encompassing key areas such as screening, automated assessment of scoliosis-related parameters, therapeutic decision-making, surgical assistance, and prognosis prediction. The review offers a broad and well-structured 360° perspective on how AI technologies can be integrated across various stages of scoliosis management. While the review is informative and well-documented, it appears to follow a narrative format and does not detail adherence to the rigorous methodology typically expected in systematic reviews. This limits the ability to assess the completeness, reproducibility and risk of bias assessment of the literature selection process.

Li et al. (9) focus on the application of machine learning methods to predict curve progression in AIS, aiming to identify patients at high risk and enable timely, proactive interventions while minimizing unnecessary exposure to ionizing radiation for low-risk individuals. The authors highlight several advantages of AI-based approaches over traditional methods, including the ability to integrate heterogeneous data sources, deliver more precise and individualized predictions, and allow for continuous model updates. However, significant limitations remain. Many models function as “black boxes,” making their outputs difficult for clinicians to interpret and potentially hindering clinical adoption. Additional concerns include the limited availability and quality of training data, the narrow focus on specific AIS subgroups—reducing the generalizability of results—and the current lack of long-term outcome studies to validate model performance over time.

Zhu et al. (10) conducted a meta-analysis to evaluate the performance of DL algorithms for automated Cobb angle estimation using radiographic images. DL algorithms performance was found to be promising, with an overall estimated CMAE of 2.99°, a level of accuracy considered acceptable by spine specialists. Among the different approaches, segmentation-based methods achieved higher accuracy (CMAE of 2.40°) compared to landmark-based techniques (CMAE of 3.31°). Despite these advances, automated Cobb angle estimation has not yet been widely adopted in routine clinical practice. Several limitations remain, notably the inconsistent consideration of end vertebra selection accuracy and clinical relevance. Furthermore, the absence of a standardized evaluation framework for Cobb angle measurement complicates the comparison and benchmarking of different algorithms. The authors also highlight the need for large-scale validation studies to support the clinical translation of these models.

As shown in Table 2, the overall confidence in the results is rated as high for the meta-analysis conducted by Zhu et al. (10), indicating a robust methodological approach and consistent findings across the included studies. In contrast, the remaining reviews exhibit only moderate or low confidence ratings, primarily due to methodological limitations, lack of standardized evaluation frameworks, and variability in study quality. This disparity in confidence levels raises important concerns regarding the reliability and generalizability of their conclusions. It also underscores the need for more rigorous study designs, transparent reporting practices, and comprehensive validation efforts in future research to strengthen the evidence base for automated Cobb angle estimation using deep learning algorithms (4).

### 4.1 Limitations

This study possesses multiple limitations that must be acknowledged when evaluating its results.

This study was performed as a quick review, which necessarily entails methodological simplification relative to a comprehensive systematic review. While the PRISMA-RR standards were adhered to and essential databases were examined, restricting the search to three databases and English-language publications may have led to the exclusion of pertinent research, thereby creating selection bias. This review exclusively examined published reviews, including systematic reviews, meta-analyses, narrative reviews, and scoping reviews, rather than primary studies. Thus, the results rely on the methodological rigor and reporting requirements of the incorporated reviews. The AMSTAR 2 evaluation indicated variety in quality, with numerous reviews lacking established processes, thorough risk-of-bias assessments, or sufficient consideration of heterogeneity (4).

Third, significant variation was noted among the included evaluations regarding artificial intelligence models, data sources, input factors, outcome measures, and performance indicators. This variability hinders direct comparisons among studies and prevents rigorous quantitative synthesis, therefore limiting the generalizability of the results. Moreover, several AI models discussed in the provided reviews lacked external validation with independent datasets. Much research relied on retrospective, single-center data, frequently characterized by small sample numbers, which heightens the danger of overestimating model efficacy and constrains real-world clinical relevance (14).

A significant restriction is the irregular incorporation of essential clinical data, including indications of skeletal maturity and psychological aspects, into predictive models. Moreover, numerous deep learning methodologies operate as “black box” systems with restricted interpretability, thereby undermining clinician trust and obstructing integration into collaborative clinical decision-making.

The lack of standardized clinical implementation guidelines, restricted interoperability with current health information systems, and insufficient evidence concerning clinical impact and cost-effectiveness continue to pose substantial obstacles to the integration of AI-based tools into standard care for adolescent idiopathic scoliosis (15).

Despite the growing body of literature on the application of artificial intelligence (AI) in scoliosis care, several critical limitations persist, currently impeding its routine clinical implementation. One of the foremost concerns is accuracy. While some AI models demonstrate promising performance in tasks such as Cobb angle estimation or vertebral landmark detection, many still lack the level of precision required for safe and effective clinical decision-making (16). This raises the risk of misdiagnoses or diagnostic omissions, particularly in borderline or complex cases.

In our rapid review, we identified several key issues across studies: insufficient external validation, absence of clinical implementation guidelines, limited incorporation of skeletal maturity indicators, and the opaque nature of many AI models.

### 4.2 Future research

Looking ahead, several priorities must be addressed to enable the responsible and effective integration of AI into scoliosis management. Chief among these is the need to improve model generalizability. This will require the development of large, diverse, and multi-center datasets that reflect the full clinical spectrum of scoliosis, including varying curve patterns, severities, and patient demographics (16).

Equally important is the rigorous validation of AI models across independent datasets and real-world clinical environments. Future research should emphasize interdisciplinary collaboration among spine specialists, data scientists, software engineers, and ethicists.

Multimodal data integration represents another critical research frontier. Incorporating imaging data alongside electronic health records, free-text clinical notes via natural language processing, and wearable sensor outputs can create more holistic and personalized AI models. Additionally, mechanisms for dynamic model updating will be necessary to ensure continued accuracy as clinical practices evolve.

## 5 Conclusion

The ultimate goal is for AI tools to serve as clinical decision support systems to provide automate measurements, improve classification, and, crucially, predict progression and treatment outcomes using a variety of models and data for personalized scoliosis treatment. Our rapid review suggests that despite promising advances in AI to improve AIS diagnosis, management and prognosis, systematic reviews often show high heterogeneity and variable level of confidence in their results. Automated Cobb angle measurement with AI has shown a relatively high accuracy. Combining deep learning models with clinical data may transform future practice, but external validation and clinical integration must be strengthened to enable effective implementation.
